# A bacterial volatile signal for biofilm formation

**DOI:** 10.15698/mic2015.10.233

**Published:** 2015-09-23

**Authors:** Yun Chen, Kevin Gozzi, Yunrong Chai

**Affiliations:** 1 Department of Biology, Northeastern University, Boston, Massachusetts, USA.; 2 Institute of Biotechnology, Zhejiang University, Hangzhou, China.

**Keywords:** volatiles, acetic acid, biofilm formation, Bacillus subtilis

## Abstract

Bacteria constantly monitor the environment they reside in and respond to potential changes in the environment through a variety of signal sensing and transduction mechanisms in a timely fashion. Those signaling mechanisms often involve application of small, diffusible chemical molecules. Volatiles are a group of small air-transmittable chemicals that are produced universally by all kingdoms of organisms. Past studies have shown that volatiles can function as cell-cell communication signals not only within species, but also cross-species. However, little is known about how the volatile-mediated signaling mechanism works. In our recent study (Chen, *et al.* mBio (2015), 6: e00392-15), we demonstrated that the soil bacterium *Bacillus subtilis* uses acetic acid as a volatile signal to coordinate the timing of biofilm formation within physically separated cells in the community. We also showed that the bacterium possesses an intertwined gene network to produce, secrete, sense, and respond to acetic acid, in stimulating biofilm formation. Interestingly, many of those genes are highly conserved in other bacterial species, raising the possibility that acetic acid may act as a volatile signal for cross-species communication.

The soil bacterium *Bacillus subtilis* serves as a model system for studies on biofilm formation. Wild strains of *B. subtilis* are capable of forming robust biofilms both under laboratory conditions and in natural settings. An interesting and puzzling observation arising in our earlier studies was that the timing of robust biofilm formation by *B. subtilis* can be clearly influenced by the presence of neighboring cells, even when the two populations of cells are physically separated from each other. We therefore speculated that the neighboring cells produced airborne signals acting to stimulate biofilm formation by the nearby *B. subtilis* cells. Earlier work in the field already showed that *B. subtilis* was able to produce a mixture of different kinds of volatiles. Some of those volatiles were shown to be involved in *B. subtilis*-plant interactions. Our own investigation revealed that acetic acid is a particularly potent volatile in stimulating biofilm formation in *B. subtilis*. When pure acetic acid was added in proximity to, but physically separated from, *B. subtilis* cells, it triggered early and robust biofilm formation by those cells.

Our published (and unpublished) data suggest that *B. subtilis* cells under biofilm induction experience a major metabolic shift toward production of small fermentation products such as acetic acid, lactic acid, ethanol, acetoin, butyric acid, etc., many of which are indeed volatiles. Genome-wide transcriptome analysis further revealed that many of the genes in the metabolic pathways for production of those fermentation products were strongly up-regulated, ranging from a few up to 100 folds (our unpublished results). Apart from that, genetic evidence implied that volatiles produced by the genetic mutants of *B. subtilis* deficient in acetic acid production failed to elicit strong biofilm-stimulating effect, again suggesting a predominant role of acetic acid as a potent volatile signal. We also observed that the genetic mutants themselves showed a delay in biofilm formation. Therefore, acetic acid may also acts as a biofilm-stimulating molecule through a canonical quorum-sensing-like mechanism. In our recent study, we mainly investigated acetic acid and characterized it as being particularly potent in stimulating biofilm formation, however, it is possible that other volatile chemicals may have similar stimulatory effects on biofilm formation or other physiological processes of the cells. For example, in addition to acetic acid, propionic acid was also shown to be able to stimulate biofilm formation in *B. subtilis* as an air-borne signal.

Acetic acid is a common metabolite derived from cell central metabolism pathways in bacteria (Fig. 1). Of note, although the genetic pathway involved in acetic acid production has been well studied in various bacteria, including *B. subtilis*, it is still unclear how transportation of this small molecule occurs in the bacterial cells and whether there is a dedicated transportation system for acetic acid to pass the cell membrane. An earlier study in *Escherichia coli* suggested that a two-gene operon, *yjcH-yjcG*, encoding a putative sodium-dependent symporter, is involved in acetic acid transportation. We identified homologous genes of *yjcH-yjcG* in *B. subtilis* and showed that the genetic mutant of the two genes in *B. subtilis* was partially defective in acetic acid transportation (Fig. 1). More importantly, the mutant displayed an early, robust biofilm phenotype compared to that of the wild type, suggesting that the rate of biofilm formation is faster in the mutant. As another piece of evidence, matrix genes were highly upregulated in the mutant. In *B. subtilis*, those matrix genes are involved in producing the sugar and protein components of the biofilm matrix that allows individual cells in the biofilm to stick to each other. We hypothesized that the mutation in the putative acetate transportation genes may result in premature accumulation of acetate inside the cell, and therefore stimulate early and robust biofilm formation. Interestingly, this operon is also conserved in many other bacteria, both Gram-negative and Gram-positive species.

**Figure 1:**
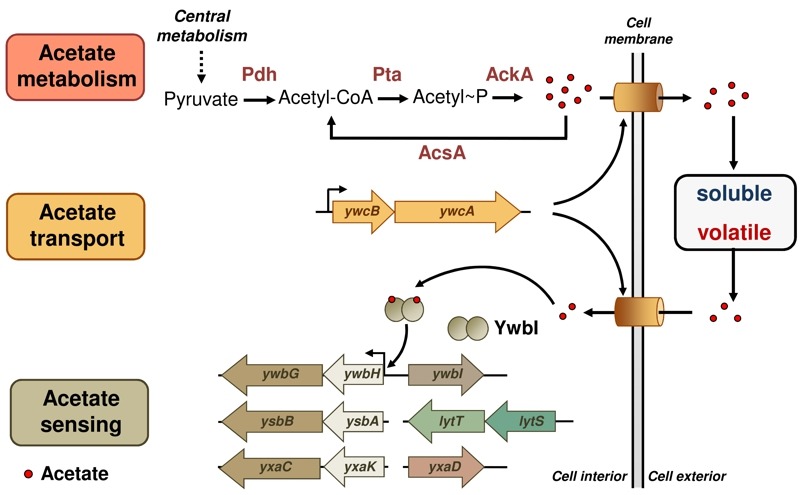
FIGURE 1: A schematic picture of the acetate signaling network in the bacterium *Bacillus subtilis*. Genes or proteins involved in acetate metabolism (Pdh, Pta, AckA, and AcsA), transport (*ywcB-ywcA*), and sensing (*ywbGHI*, *ysbBA-lytTS*, and *yxaCKD*) are described. Secreted acetate can act as either a volatile signal or a canonical quorum-sensing-like signal in solution.

Another pivotal question is how acetic acid is sensed by *B. subtilis* and how cells respond to it in stimulating biofilm formation. An important category of sensory proteins in bacteria consists of transcription factors, which directly bind to small molecules and subsequently regulate gene expression. For acetic acid, one such example was previously characterized in the Gram-positive bacterium *Staphylococcus aureus*, in which a transcription factor CidR directly binds to acetic acid and activates genes encoding holin-antiholin-like proteins. Originally identified in bacterial phages, those proteins are involved in host cell lysis and release of phage particles. It is now believed that many bacterial genomes also contain similar genes and the protein products of those genes play an important role in programmed cell death by modulating the activities of bacterial cell lysis enzymes, also known as murein hydrolases. In *S. aureus*, accumulation of acetic acid may act as a signal to trigger localized cell death and release of extracellular DNA (eDNA), which turns out to be an important step during *S. aureus *biofilm development.

Interestingly, these holin-antiholin-like genes are highly conserved in *B. subtilis* (*ywbGH*, *ysbBA*, and *yxaCK*, Fig. 1). We applied multiple approaches by performing genetic mutations, overexpression of specific genes, and various bioassays, and concluded that a similar acetic-acid-responsive pathway is also present in *B. subtilis* (and likely in other Gram-positive bacteria as well). We observed that those genes encoding holin-antiholin-like proteins were strongly activated in the presence of acetic acid. We also showed that some of those genes are directly involved in biofilm formation. As a possible mode of action for those holin-antiholin-like proteins in *B. subtilis*, they may be involved in programmed cell death and release of eDNA, similar to what was proposed in *S. aureus*.

Our evidence suggests that acetic acid may have a second mode of action in stimulating biofilm formation in *B. subtilis*. Acetic acid also directly activates matrix genes, although the underlying mechanism is still unclear. It is worth pointing out that the metabolic pathway of acetic acid also involves intermediate products such as acetyl-CoA and acetyl-phosphate. Acetyl-phosphate has been shown to be another central metabolic signal that can directly involve protein phosphorylation or acetylation, two very important modifications for response regulators. Past studies suggested that Spo0A and DegU, two important response regulators and key regulators for biofilm formation in *B. subtilis,* can be phosphorylated by free acetyl-phosphate. It will be interesting to see whether intermediate products of acetate metabolism, such as acetyl-phosphate, can be an important secondary messenger for bacterial multicellular development or not.

Thinking of small volatile chemicals such as acetic acid from a broader point of view, those chemicals are not only produced by bacterial cells, but also by eukaryotic cells. There are a number of published reports that gut microbial species are able to produce small metabolites to modulate human immune system. Recent studies also demonstrated that volatile molecules produced by bacteria are able to stimulate plant growth and acquired systemic resistance.

In summary, cell-cell communication and interaction through small volatile signals have been an understudied research field. With increasing evidence that those volatile molecules are not only metabolically important for shaping the physiology of individual cells, but also important as signal molecules to regulate multicellular process such as biofilm formation, as well as bacteria-host interactions, further work will need to be done to improve our understandings of those signaling mechanisms at the molecular level.

